# Flow does not alter eNOS phosphoryation at Ser1179 or Thr495 in preconstricted mouse mesenteric arteries

**DOI:** 10.14814/phy2.13864

**Published:** 2018-09-10

**Authors:** Robin C. Looft‐Wilson, Sarah E. Todd, Kristen M. Berberich, Madeline R. Wolfert

**Affiliations:** ^1^ Department of Kinesiology and Health Sciences The College of William & Mary Williamsburg Virginia

**Keywords:** Endothelium, flow‐induced vasodilation, phenylephrine, shear stress

## Abstract

In arteries, endothelium‐dependent vasodilatory agonists and flow‐induced shear stress cause vasodilation largely by activation of the endothelial enzyme eNOS, which generates nitric oxide that relaxes vascular smooth muscle. Agonists activate eNOS in part through increased phosphorylation at Ser1179 and decreased phosphorylation at Thr495. We previously found that preconstriction of intact, isolated mouse mesenteric arteries with phenylephrine also caused increased Ser1179 and decreased Thr495 eNOS phosphorylation, and sequential treatment with the vasodilatory agonist acetylcholine did not cause any further change in phosphorylation at these sites, despite producing vasodilation. The present study tests the hypothesis that luminal flow in these arteries preconstricted with phenylephrine also produces vasodilation without phosphorylation changes at these sites. First‐order mesenteric arteries, isolated from male C57/BL6 mice (7–20 weeks of age) anesthetized with pentobarbital (50 mg/kg, i.p.), were cannulated, pressurized, and treated with stepped increases in luminal flow (15–120 *μ*L/min). Flow resulted in dilation that plateaued at ~60 *μ*L/min (31.3 ± 3.0% dilation) and was significantly (*P* < 0.001) NOS‐dependent at all flow rates (determined by 10^−4^ mol/L L‐NAME treatment). In separate arteries, preconstriction with phenylephrine (10^−5^ mol/L) resulted in increased eNOS phosphorylation at Ser1179 (*P* < 0.05) and decreased phosphorylation at Thr495, but subsequent flow at 60 *μ*L/min for 5 or 15 min did not cause further changes in phosphorylation, despite causing dilation. Thus, flow‐induced dilation does not require changes in these eNOS phosphorylation sites beyond those induced by alpha_1_‐adrenergic stimulation with phenylephrine, indicating that eNOS is activated by other mechanisms during acute flow‐induced dilation of preconstricted arteries.

## Introduction

Many arteries in the body dilate in response to increased blood flow, such as during exercise or after acute ischemia (Markos et al. 2013; Green et al. 2014, 2017). Increased flow causes shear stress on the endothelial cells that line the artery, and the resulting vasodilation is due at least in part to activation of the endothelium enzyme, eNOS, which generates nitric oxide and causes smooth muscle relaxation (Gilligan et al. 1994; Dimmeler et al. 1999; Boo and Jo 2003; Sessa 2004; Goto et al. 2007; Balligand et al. 2009; Green et al. 2014; Casey et al. 2017). eNOS is activated by increased endothelial cell calcium and phosphorylation at one or more sites, although the relative importance of each of these events to shear stress‐induced activation is not clear (Dimmeler et al. 1999; Boo and Jo 2003; Sessa 2004; Balligand et al. 2009; Rafikov et al. 2011). Endothelium‐dependent vasodilatory agonists, such as actylcholine, generally cause increased eNOS phosphorylation at Ser1179 and decreased phosphorylation at Thr495, and both of these events promote eNOS activation (Sessa 2004; Rafikov et al. 2011). Ser1179 is also a prominent eNOS phosphorylation site during acute shear stress‐induced eNOS activation. This phosphorylation event has been identified primarily in flow‐treated cultured endothelial cells (Dimmeler et al. 1999; Boo et al. 2002b; Li et al. 2004), with evidence also in isolated, perfused rat mesenteric vascular bed (Figueroa et al. 2013). The role of Thr495 during acute shear stress, however, is unclear, given that both no change (Boo et al. 2002a) and increased phosphorylation (Barauna et al. 2013) of this site have been reported in cultured endothelial cells.

This purpose of this study was to determine whether Ser1179 is phosphorylated and Thr495 is dephosphorylated on eNOS in intact mesenteric arteries in vitro during acute flow‐induced vasodilation, specifically during preconstriction with the alpha_1_‐adrenergic agonist phenylephrine. The purpose of phenylephrine preconstriction was to induce tone in an artery with little myogenic tone so that vasodilation could be observed, and to mimic the tonic sympathetic vasoconstriction experienced by this (and many arteries) in vivo (Abu‐Amarah et al. 1998). In addition, we previously found that preconstriction of mouse mesenteric arteries with phenylephrine caused increased eNOS phosphorylation at Ser1179 and decreased phosphorylation at Thr495 (Looft‐Wilson et al. 2013), and cumulative addition of acetylcholine (after phenylephrine preconstriction) caused NOS‐dependent vasodilation but no further changes in eNOS phosphorylation at these two sites. This suggests that phosphorylation changes at one or both of these sites is permissive for acetylcholine‐induced NOS activation in these conditions, but is not the primary driver. Therefore, in the present study, we sought to determine whether the same was true for flow‐induced vasodilation in preconstricted arteries.

Treatment of arteries with phenylephrine has been shown to activate NOS and attenuate the phenylephrine‐induced constriction through a process termed “myoendothelial feedback” (Dora et al. 1997; Kerr et al. 2012, 2015). Myoendothelial feedback involves IP_3_ flux from smooth muscle to endothelial cells through myoendothelial gap junctions, and subsequent endothelial hyperpolarization and localized increased intracellular calcium leading to eNOS activation and smooth muscle relaxation (Dora et al. 2000; Jackson et al. 2008; Nausch et al. 2012; Tran et al. 2012; Kerr et al. 2015). We were the first to show that phenylephrine also caused eNOS phosphorylation changes at Ser1179 and Thr495 and it is likely that these changes are participating in its activation (Looft‐Wilson et al. 2013). However, it is not yet known what kinase/s is responsible for these changes.

The present study shows that there is no change in eNOS phosphorylation at Ser1179 or Thr495 in phenylephrine‐constricted mouse mesenteric arteries during flow‐induced vasodilation in vitro, beyond the changes induced by phenylephrine alone. Together with our previous study (Looft‐Wilson et al. 2013), this study shows that mesenteric arteries preconstricted with a sympathetic agonist, vasodilate in response to acetylcholine or flow in a largely NOS‐dependent manner that does not require additional phosphorylation changes at these two key eNOS phosphorylation sites.

## Materials and Methods

### Animals

All experimental procedures were approved by the William & Mary Institutional Animal Care and Use Committee and are in accordance with the Guide for the Care and Use of Laboratory Animals (National Research Council). Male C57BL/6 mice (*n* = 101) at least 3 weeks of age (obtained from Charles River Laboratories, Inc., Wilmington, MA) were housed in standard cages in a climate‐controlled, 12‐h light/12‐h dark environment, with free access to food and water. At age 7–20 weeks, mice were anesthetized with sodium pentobarbital (Nembutal, 50 mg/kg body weight intraperitoneal) during the day (between 10 am and 4 pm), and anesthesia was confirmed by absence of toe‐pinch and corneal reflexes. Pentobarbital was chosen because it is an approved anesthetic method, fast‐acting, reliable, and commonly used in isolated vessel studies (Loufrani et al. 2002, 2008; Figueroa et al. 2013; Ashley et al. 2018). The dose of pentobarbital was chosen to be the minimal recommended dose to achieve the anesthetic plane. When the anesthetic plane was reached, the intestines were immediately excised and placed in cold physiological salt solution (PSS; in mmol/L: 145 NaCl, 4.7 KCl, 2.0 CaCl_2_, 1.17 MgSO_4_, 1.2 NaH_2_PO_4_, 2.0 MOPS, 0.02 EDTA, 5.0 glucose, 2.0 pyruvate, pH 7.4), and first order mesenteric arteries were removed for artery function (flow‐induced dilation) and eNOS phosphorylation experiments. Euthanasia resulted from exsanguination.

### Mesenteric artery pressure myography

One or two‐first‐order mesenteric arteries were isolated from each mouse. Arteries were cannulated in a vessel chamber (Danish Myo Technology, Aarhus, Denmark), filled with PSS, secured with nylon suture (8–0, S & T, Neuhausen, Switzerland), gently cleared of blood, and pressurized to 75 mmHg by perfusion with PSS + 1% BSA (or 3% BSA in some experiments). The cannula resistances were matched by ensuring that onset and cessation of fluid streams through the cannula were at the same pressures (measured in both cannula simultaneously). The chamber was mounted on a microscope stage (Danish Myo Technology), continuously superfused with fresh PSS, and equilibrated at 37°C for 30 min with no luminal flow. The superfusion solution and vessel chamber were open to the ambient air. Luminal diameter was measured using a 10× objective, CCD camera, and either VediView Software (Danish Myo Technology) or IonOptix software (Milton, MA).

### Flow‐induced dilation responses

Individual 1^st^ order mesenteric arteries were collected from 46 mice and prepared as described in the previous section. Arteries were preconstricted to phenylephrine (10^−5^ mol/L, in superfusate, 100 mL recirculated) for 10 min. This dose of phenylephrine was chosen because it consistently produces a deep constriction in our set‐up of ~50% of maximal diameter (Looft‐Wilson et al. 2008), which would allow for detection of the largest magnitude of flow‐induced dilation. Flow was increased in steps (0, 15, 30, 60 *μ*L/min; and 120 *μ*L in some arteries) for 5 min each by raising the inflow and lowering the outflow reservoirs equal distance. Flow was measured using a manual flowmeter (Gilmont Instruments, #GF‐3060) or digital flowmeter (Myoflow, DMT) in series with the outflow cannula. Inflow and outflow pressures were measured with in‐line pressure transducers and a pressure monitor (Pressure Monitor 4, Living Systems, Burlington, VT) to ensure that average pressure of 75 mmHg was maintained. This was followed by addition of acetylcholine (10^−5^ mol/L, in superfusate, 100 mL recirculated, containing phenylephrine) for 5 min to confirm endothelial cell integrity. Vasomotor responses were measured with or without L‐NAME (10^−4^ mol/L) (added to superfusate in all, and perfusate in some, starting at equilibration) to block nitric oxide synthases. At the end of each experiment, arteries were superfused with Ca^++^‐free PSS + EGTA (1 mmol/L) to determine maximal diameter. Of the 46 arteries tested, 10 were eliminated from the analysis due to technical failure (i.e., failure to maintain pressure, flow, or temperature; *n* = 6) or poor responsiveness to phenylephrine (*n* = 1) or acyetylcholine (*n* = 3). Artery responses to 1% versus 3% BSA perfusate were not different (Fig. S1A and B), with the exception of the response to the highest flowrate in L‐NAME treated arteries (Fig. S1B), so the data were combined. Adding L‐NAME to both the perfusate and superfusate did not affect the responses to flow (Fig. S1C), so all arteries were combined.

### Artery treatment for determination of eNOS phosphorylation during flow‐induced dilation

Arteries were cannulated as described above (using 1% BSA perfusate) and treated with agonists and/or flow (described below) then immediately frozen in liquid N_2_ and stored at −85°C for use in immuno‐blot determination of eNOS phosphorylation. All arteries underwent 30 min equilibration followed by one of the following treatments: (1) “No Treatment”: 25 min incubation to serve as a time control, (2) “PE”: constriction in phenylephrine (10^−5^ mol/L) for 10 min, (3) “ACh”: constriction in PE for 10 min, followed by 5 min with acetylcholine (10^−4^ mol/L), (4) “5 min Flow”: constriction in PE for 10 min, followed by 5 min at ~60 *μ*L/min flow, or (5) “15 min Flow”: constriction to PE for 10 min, followed by 15 min at ~60 *μ*L/min flow. Diameter responses were measured in each treatment. A total of 108 arteries were collected from 55 mice (two arteries were collected from most mice) and underwent treatment, with different treatments given to artery pairs collected from the same mouse. A total of 86 arteries were used in the immuno‐blot analysis, and arteries that were damaged due to technical error and/or poorly responsive (*n* = 13), or that were overrepresented in a treatment group (*n* = 9) were not used.

### eNOS phosphorylation

Immuno‐blotting was performed to measure the relative eNOS phosphorylation at Ser117 or Thr495 to total eNOS (four or six pooled first order arteries for each sample) as previously described (Looft‐Wilson et al. 2013). Tissues were homogenized in 50 or 80 *μ*L of lysis buffer with phosphatase inhibitors (50 mmol/L Tris–HCl, 100 mmol/L NaF, 15 mmol/L Na_4_P_2_O_7_, 1 mmol/L Na_3_VO_4_, 1% Triton X‐100, and 1:200 protease inhibitor cocktail solution (#P2714; Sigma, St. Louis, MO); pH = 7.6), incubated for 1 h at 4°C, and centrifuged (12,100 *g*, 10 min) to remove insoluble material. Proteins were separated by 10% SDS‐PAGE (4% stacking gel), then electro‐transferred to a nitrocellulose membrane. Total protein was visualized on the membrane with ponceau‐S to ensure effective transfer. Membranes were first immuno‐labeled for phosphorylated eNOS, either pSer1179‐eNOS (1:750; BD Biosciences, San Jose, CA) or pThr495‐eNOS (1:1000; BD Biosciences), followed by horseradish peroxidase‐conjugated secondary antibody (anti‐mouse 1:5000; Pierce Biotechnology, Rockford, IL), and visualized with enhanced chemiluminescence (Pierce Biotechnology) captured on film. The membrane was then stripped for 20 min (Restore Western Blot Stripping Buffer, Thermo Scientific, Waltham, MA), and reprobed with eNOS (1:1000; BD Biosciences) using the same procedure as with the phosphorylated forms. Films were electronically scanned and protein band density was quantified using IMAGE J (NIH).

### Data analysis

**Figure 1:**
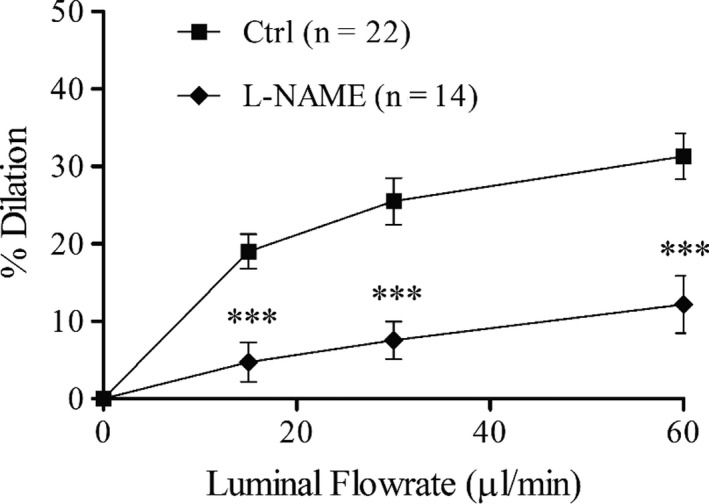
Dilation responses of mouse mesenteric arteries to step increases (5 min each) in luminal flow with or without NOS blockade (L‐NAME, 10^−4^ mol/L). ***P <* *0.001 compared to control (two‐way ANOVA). Arteries were preconstricted with phenylephrine (10^−5^ mol/L) before and during flow. Vessel diameters and responses to agonists are shown in Table 1. Calculated shear stresses at each flowrate is shown in Table 2.

**Figure 2:**
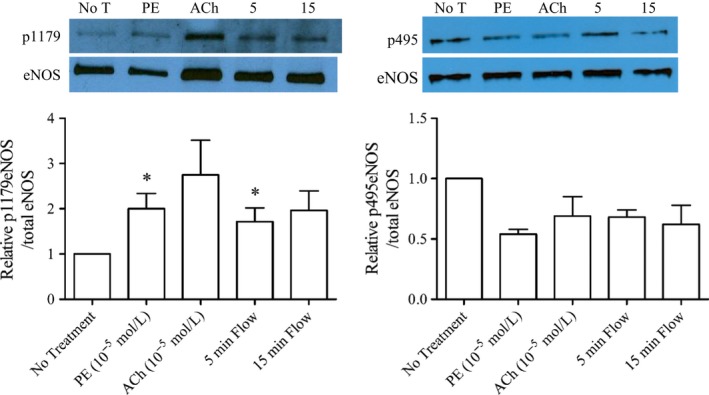
eNOS Phosphorylation responses with various treatments in isolated, cannulated mesenteric arteries. Four to six vessels were treated and pooled for each sample for each treatment. Representative immuno‐blots are shown above each quantification graph. (A) Relative ratio of phosphorylation of eNOS at Ser1179/total eNOS. Each treatment group represents four sample sets (over 4 immuno‐blots), except “5 min Flow” which represents three sample sets. *Significantly greater (*P* < 0.05) compared to “No Treatment” (Student's *t*‐test). One‐way ANOVA did not show any significant differences between the groups. (B) Relative ratio of phosphorylation of eNOS at Thr495/total eNOS. Each treatment group represents two sample sets (over 2 immuno‐blots). Statistical comparisons were not performed due to the small number of sample sets. All immuno‐blot images are presented in Figure S4. Vessel responses for each groups used in each blot are shown in Table 3 [Color figure can be viewed at wileyonlinelibrary.com].

Statistics were performed using Prism 4 software (GraphPad Software, Inc., San Diego, CA) (ANOVAs) or Excel (Microsoft, Redmond, WA) (Student's *t*‐tests). Artery diameters and responses (Table 1) were compared between control and L‐NAME by two‐tailed Student's *t*‐test. % Dilation (Fig. 1) was calculated: [(diameter after acetylcholine addition or at each flow step − preconstricted diameter)/(maximal diameter − preconstricted diameter)] × 100. Preconstricted diameter is the diameter 10 min after addition of phenylephrine. Shear stress (dynes/cm^2^) was calculated by: (4 × viscosity × flow × 10^9^/*π* × radius^3^); with viscosity = 0.008 dynes × sec/cm^2^ for PSS with BSA (Kuo et al. 1995), flow in *μ*L/sec, the 10^9^ factor to correct for using *μ*L/sec for flow (1 *μ*L = 10^9^ *μ*m^3^), and radius in *μ*m (Learmont et al. 1996). % Dilation responses with and without L‐NAME were compared by two‐way ANOVA with Bonferroni post hoc tests. Calculated shear stress was compared at each step increase in flow at the initiation of flow (initial) versus after 5 min equilibration (final), between flowrates at initial and final, and with L‐NAME at initial and final by ANOVAs with Bonferroni post hoc tests (Table 2). Relative fraction of phosphorylated eNOS was determined by dividing the signal (band density) for pSer1179eNOS or pThr495eNOS by the total eNOS signal, then normalizing each ratio to the “No Treatment” ratio to allow averaging across membranes (Fig. 2, Fig. S4). Relative phosphorylation was compared to “No Treatment” by *t*‐test and between all treatments by ANOVA. Artery responses between sets of arteries used in the immunoblots (Table 3) were compared by ANOVA. Significance was at *P* < 0.05.

**Table 1:** Artery diameters and responses (mean ± SEM)

	Control (*n* = 22)	L‐NAME (*n* = 14)
Baseline (*μ*m)	248 ± 8	210 ± 12^a^
PE (10^−5^ mol/L) (*μ*m)	101 ± 5	65 ± 2^b^
ACh (10^−5^ mol/L) (*μ*m)	210 ± 9	130 ± 14^b^
Maximal (*μ*m)	257 ± 6	242 ± 9
Basal tone (% of maximal)	96.4 ± 1.5	86.6 ± 2.8^a^
PE response (% of maximal)	39.4 ± 1.8	27.4 ± 1.7^c^
ACh response (% of maximal)	81.7 ± 2.7	52.8 ± 4.8^b^

Maximal diameter was measured at end of experiment with Ca^++^‐free PSS + 1 mmol/L EGTA. Baseline diameter was measured after 30 min equilibration, at beginning of experiment.

a Significantly different from Control, *P* < 0.01 (Student's *t*‐test).

b Significantly different from Control, *P* < 0.0001 (Student's *t*‐test).

c Significantly different from Control, *P* < 0.001 (Student's *t*‐test).

**Table 2:** Calculated shear stress with each step increase in flow (mean ± SEM)

	Initial shear stress (dynes/cm^2^)	Final shear stress (dynes/cm^2^)
Control (*n* = 22)		
15 *μ*L/min	24.9 ± 2.7	11.1 ± 1.1
30 *μ*L/min	22.1 ± 2.2	17.8 ± 1.7
60 *μ*L/min	35.7 ± 3.3	28.9 ± 2.7
L‐NAME (*n* = 14)		
15 *μ*L/min	80.5 ± 6.8	64.1 ± 8.1
30 *μ*L/min	128.3 ± 16.1^a^	105.1 ± 14.0^a^
60 *μ*L/min	210.2 ± 28.0^a^, ^b^	190.3 ± 34.1^a^, ^c^

Initial shear stress is determined at initiation of each flow step. Final shear stress is determined after 5 min equilibration at each flow step. There were no differences in initial versus final shear stress at any flow step.

a Significantly different from equivalent Control flowrate, *P* < 0.001 (ANOVA).

b Significantly different from L‐NAME: 15 *μ*L/min, *P* < 0.001 (ANOVA).

c Significantly different from other flowrates treated with L‐NAME, *P* < 0.001 (ANOVA).

**Table 3:** Responses (mean ± SEM) of arteries in each treatment group used in immuno‐blots

	Set 1 (*n* = 4 each)	Set 2 (*n* = 4 each)	Set 3 (*n* = 4 each)	Set 4 (*n* = 6 each)	Combined (*n* = 14–18 each)
No treatment: final diameter (*μ*m)	220 ± 10	221 ± 7	231 ± 15	210 ± 12	219 ± 6
PE: final diameter (% of max)	29.3 ± 1.8	40.2 ± 2.8	31.7 ± 2.5	37.1 ± 3.6	34.8 ± 1.7
ACh: final diameter (% of max)	85.3 ± 9.6	75.6 ± 10.8	88.2 ± 5.2	80.6 ± 2.4	82.2 ± 3.3
5 min flow % dilation	–	22.2 ± 8.3	20.7 ± 7.4	14.4 ± 1.4	18.4 ± 3.1^a^
Initial shear stress (dynes/cm^2^)	–	220.5 ± 136.6	159.4 ± 33.7	78.3 ± 18.1	142.1 ± 40.4
Initial shear stress (dynes/cm^2^)	–	39.4 ± 2.1	60.6 ± 15.1	47.5 ± 11.9	48.9 ± 6.6
15 min flow% dilation	31.6 ± 4.6	42.9 ± 4.6	34.5 ± 3.9	29.5 ± 6.5	34.1 ± 2.8
Initial shear stress (dynes/cm^2^)	100.4 ± 27.6	257.0 ± 111.5	137.0 ± 52.8	121.7 ± 18.7	150.4 ± 29.3
Final shear stress (dynes/cm^2^)	28.9 ± 2.6	26.6 ± 4.3	25.2 ± 7.5	35.8 ± 6.7	29.8 ± 3.0

No significant differences between sets. Maximal diameter (max) is estimated from baseline diameter. Diameter refers to lumen diameter.

a Significantly different from 15 min flow: % Dilation, *P* < 0.001 (Student's *t*‐test).

## Results

### Mesenteric artery flow‐induced vasodilation responses

Stepped increases in flow caused progressive dilation that reached 31.3 ± 3.0% at the maximal flow of 60 *μ*L/min (Fig. 1). This dilation response appears to be maximal because a further increase in flow (performed in some arteries) resulted in little additional dilation (Fig. S2). NOS blockade (L‐NAME, 10^−4^ mol/L) significantly decreased the dilation response at all flowrates and decreased the maximal dilation to 10.3 ± 3.4%, indicating that nitric oxide synthases are important for the flow‐induced dilation in this artery under these conditions. Treatment with L‐NAME resulted in significant basal tone, smaller diameter with phenylephrine treatment, and decreased dilation to acetylcholine, which are all expected with NOS blockade (Table 1). The smaller diameter with phenylephrine preconstriction in L‐NAME‐treated arteries and impaired dilation to flow resulted in significantly greater calculated shear stress with higher flowrates (30 and 60 *μ*L/min) at the point of flow initiation (Initial Shear Stress) and after equilibration (Final Shear Stress) compared to Control (Table 2). Although the final shear stress was less than the initial shear stress with each increase in flowrate due to artery dilation, it was not significant for any flowrate in either the Control of L‐NAME groups (Table 2). In the L‐NAME group the initial and final shear stress at the highest flowrate (60 *μ*L/min) was significantly greater than at one or more of the lower flowrates due to the reduced dilation response (Table 2).

### Mesenteric artery eNOS phosphorylation during flow‐induced dilation or treatment with agonists

Separate arteries underwent no treatment (time control), or were treated with phenylephrine, phenylephrine plus flow (60 *μ*L/min to induce maximal dilation), or phenylephrine plus acetylcholine (as described in Methods). Treatment with flow for 5 min resulted in dilation (18.4 ± 3.1% dilation [Table 3]) that was significantly lower than the dilation in arteries treated with flow for 15 min (34.1 ± 2.8% dilation [Table 3]). Dilation to 15 min flow is similar to the maximal response of arteries used in the stepped‐flow experiments (31.3 ± 3.0% dilation [Fig. 1]; compared by Student's *t*‐test), indicating that maximal flow‐induced dilation was achieved with 15 min of flow at 60 *μ*L/min. eNOS phosphorylation at Ser1179 significantly increased with PE compared to no treatment, but no further increase was observed with 5 or 15 min Flow, or ACh treatments (Fig. 2A). PE and 5 min flow were significantly greater than no treatment (*t*‐test), but other treatments were not (Fig. 2A). eNOS phosphorylation at Thr495 was measured in two blots and indicated a similar decrease in phosphorylation in all treatment groups compared to no treatment (Fig. 2B). Notably, immunoblots of sample sets #1–3 generally showed 2× or greater Ser1179 phosphorylation in each of the treatment groups, but the immunoblot of sample set #4 showed no increase in phosphorylation in the PE, ACh, or 15 min flow groups (Fig. S3), despite similar functional responses (Table 3), calculated initial and final shear stresses (Table 3), and decreased Thr495 phosphorylation (Fig. S4), leaving no justification to exclude it.

## Discussion

This study shows, for the first time, that maximal flow‐induced vasodilation can occur without an increase in eNOS phosphorylation at Ser1179, and still be largely NOS dependent. Therefore, phosphorylation at this site may be playing a permissive role, or serve to prime eNOS for activation by other mechanisms. The mechanisms by which eNOS is activated by flow are not described in this study, but could include increased calcium‐dependent activation, phosphorylation events at other sites, or changes in protein‐protein interaction or localization of the enzyme (Sessa 2004; Balligand et al. 2009; Rafikov et al. 2011). The lack of changes in both phosphorylation sites with acute flow beyond that induced by phenylephrine is consistent with the lack of increase observed with acetylcholine treatment in preconstricted arteries in our previous study (Looft‐Wilson et al. 2013), and confirmed again in this study (Fig. 2, “ACh” responses). Thus, eNOS phosphorylation changes with phenylephrine preconstriction (mimicking the sympathetic constriction prevalent in vivo in mesenteric arteries) allows subsequent NOS‐dependent vasodilation through both agonist and flow‐induced mechanisms, without additional phosphorylation changes at these sites.

This study also documents for the first time that phosphorylation at Thr495 does not change with acute shear stress beyond the decreased phosphorylation induced by phenylephrine preconstriction. To our knowledge, this is the first report of phosphorylation status of this residue in intact arteries with acute flow‐induced dilation. One study examined the chronic effects (4 h) of increased blood flow in the lamb lung on eNOS phosphorylation at Thr495 and found it was increased (Kajikawa et al. 2015). Thus, it is likely that acute and chronic effects of shear stress on eNOS regulation are different.

The contribution of eNOS to flow‐induced vasodilation was slightly greater than 50% in this study, which is consistent with other studies in mouse mesenteric arteries in which flow‐induced vasodilation is reportedly ~50% NOS dependent (Loufrani et al. 2008; Ahn et al. 2017). The large dependence on eNOS for this response make it a good model for studying eNOS regulation during flow‐induced dilation. In addition, its status as a resistance artery makes it a relevant model for controlling blood pressure and the magnitude of blood flow to its downstream vascular bed (Christensen and Mulvany 1993). A recent study showed that flow‐induced activation of NOS in mouse mesenteric arteries requires inward rectifying potassium channels, specifically Kir2.1 (Ahn et al. 2017). The remaining flow‐induced vasodilation was attributable to activation of small conductance calcium‐activated potassium channels (Ahn et al. 2017). This means this artery uses both NOS and non‐NOS mechanisms to control flow‐induced dilation.

To our knowledge, the only other study to specifically examine flow‐induced eNOS phosphorylation in intact resistance arteries was a study performed in isolated mesenteric circulation, which found increased Ser1179 phosphorylation with acute flow (Figueroa et al. 2013). Although these findings are contrary with ours, a major difference in this study is that the arteries were not preconstricted and vasodilation was not documented. Because it is known that preconstriction increases eNOS Ser1179 phosphorylation, then arteries that are not preconstricted likely have less baseline phosphorylation and more capacity to be phosphorylated with shear stress. It is also possible that the architecture of the endothelial cell layer in a fully relaxed artery promotes different shear stress‐induced mechanotransduction than in a preconstricted intact artery, which is visibly folded, and perhaps this regulates eNOS differently. Examination of eNOS regulation in the preconstricted state is an important aspect of the present study, because this is the expected state in these arteries in vivo. Mesenteric arteries are richly innervated with sympathetic vasoconstrictor nerves (Long and Segal 2009), and are under chronic sympathetic control to maintain blood pressure (Abu‐Amarah et al. 1998), and under high sympathetic stimulation during exercise and acute cardiovascular stress (Reilly et al. 2001; Volianitis and Secher 2016). Thus, it is predicted that in this large vascular bed, flow‐induced dilation would typically occur under conditions in which the arteries are preconstricted by norepinephrine (primarily through alph_1_‐adrenonergic stimulation of smooth muscle), similar to the model used in this study. Therefore, in vivo, eNOS may be basally phosphorylated at Ser1179, and flow‐induced dilation may then rely on other mechanisms of eNOS activation in this vascular bed.

Increased eNOS phorphorylation on Ser1179 (and Ser1177 in humans) has been reported in large arteries in response to acute exercise, specifically mouse aorta, iliac and femoral artery (combined and analyzed as one sample), and human brachial artery (Zhang et al. 2009; Casey et al. 2017). Exercise, however, promotes more than just an increase in shear stress and is likely to lead to neuro‐humoral changes. For example, an increase in sympathetic outflow is well documented with exercise (Fisher et al. 2015) and was likely in the study of human brachial artery, as evidenced by the increase in heart rate (Casey et al. 2017). Thus, it is possible that the increase in phosphorylation could be due to other factors during exercise rather than the shear stress exclusively, but this is challenging to ascertain with an exercise stimulus in vivo. The present study sought to determine the specific contribution of flow‐induced shear stress to both eNOS phosphorylation and artery dilation, so while the results from these exercise studies are not directly comparable, they support the notion that this phosphorylation event occurs either during or before the flow‐induced dilation.

It is currently unknown whether preconstriction with vasoconstrictors other than phenylephrine would result in myoendothelial‐dependent eNOS phosphorylation as observed in this study, because this event has only been reported with this vasoconstrictor (Looft‐Wilson et al. 2013). It might be predicted, however, that only vasoconstrictor stimuli which result in smooth muscle IP_3_ generation may activate and/or phosphorylate NOS via myoendothelial communication because a recent study indicates that only some vasoconstrictors induce myoendothelial feedback and this ability seems to be IP_3_‐dependent (Wei et al. 2018). Specifically, it has been shown that NOS‐dependent myoendothelial feedback occurs with phenylephrine, norepinephrine, and high‐frequency perivascular nerve stimulation in rat mesenteric arteries (Kerr et al. 2015; Wei et al. 2018). Based on evidence from small arteries/arterioles of several rodent species, these stimuli act exclusively on smooth muscle through alpha_1_‐adrenergic receptor stimulation (Jackson et al. 2008; Nausch et al. 2012) and generate smooth muscle IP_3_ that stimulates endothelial calcium release, hyperpolarization, and NOS generation via myoendothelial gap junction communication (Kansui et al. 2008; Kerr et al. 2015). In contrast, neither the vasoconstrictor U46619 (a thromboxane A_2_ mimetic) nor myogenic tone induce myoendothelial feedback in rat mesenteric arteries, and these stimuli do not induce IP_3_ signaling (Wei et al. 2018). The ability of vasoconstrictors, other than phenylephrine, to phosphorylate eNOS via myoendothelial communication would need to be specifically tested in our model, and would be experimentally challenging given that some vasoconstrictors (e.g., U46619, 5‐HT) may act directly on endothelial cells to activate eNOS.

### Limitations

It should be noted that the NOS inhibitor used in this study (and nearly all available eNOS inhibitors) inhibits other NOS isoforms as well. Therefore, it is possible that nNOS and or iNOS are also involved in flow‐induced vasodilation and this blocker would not distinguish their roles. nNOS is immunohistochemically present in the endothelium of mouse mesenteric arteries (Takaki et al. 2008; Silva et al. 2016), and iNOS can be induced in endothelium with knock‐out of the other NOS isoforms (Takaki et al. 2008). Additionally, we found the presence of expression (using western blot) of both of these isoforms in whole mouse mesenteric arteries (Looft‐Wilson et al. 2017). However, there is currently no evidence that these NOS isoforms are involved in flow‐induced vasodilation in mouse mesenteric arteries. Moreover, there is evidence (using inhibitors that are more specific to nNOS) that nNOS is not involved in flow‐induced dilation in isolated mouse coronary arteries (Huang et al. 2002) or post‐ischemic flow‐mediated dilation in vivo (a more complex response) in human radial artery (Seddon et al. 2009; Shabeeh et al. 2017) or rat cerebral arterioles (Bauser‐Heaton and Bohlen 2007). Overall, there is no clear role for NOS isoforms other than eNOS to flow‐induced vasodilation in mouse mesenteric arteries or other vessels.

The present study initiated flow‐induced dilation using step increases in flow, rather than step increases in pressure gradient or shear stress. The reason for this selection is because we found that dilation responses were relatively consistent across arteries of slightly different diameters and magnitudes of preconstriction at each flow rate (Fig. 1), and these response curves were similar to those found in many previous studies using stepped increases in flow in mouse mesenteric arteries (Loufrani et al. 2002; Ohlmann et al. 2005; Sennoun et al. 2009; Priou et al. 2010; Leonetti et al. 2013; Tarjus et al. 2017; Xu et al. 2018), and more robust than found in some studies (Douglas et al. 2008; Besnier et al. 2018). Although the calculated shear stress was different between arteries at the start of flow (Initial Shear Stress), due to variation in diameters after preconstriction with phenylephrine, the final calculated shear stress (Final Shear Stress) values had much less variability as indicated by the smaller SEM (“Control” arteries in Tables 2 and 3). This may indicate that the artery is regulating its shear stress to a given target. The shear stress values in these flow experiments are in the range of those measured in vivo in large arteries of mice (Cheng et al. 2007). Notably, NOS inhibition did not allow for regulation of artery diameter to normalize shear stress, resulting in very high shear stress values with increases in flow (“L‐NAME” arteries in Table 2).

The present study used a relatively high dose of phenylephrine to induce preconstriction. This dose was chosen because it reliably produces near maximal constriction with lower variation in constriction responses than submaximal doses (Fig. 1A in [Looft‐Wilson et al. 2008]) and the deep constriction allows observation of a larger magnitude flow‐induced vasodilation response. Moreover, use of this agonist and this magnitude of preconstriction is typical when examining flow‐induced dilation in these arteries (Loufrani et al. 2002; Sennoun et al. 2009; Tarjus et al. 2017; Besnier et al. 2018; Xu et al. 2018). This phenylephrine dose results in eNOS phosphorylation changes (Looft‐Wilson et al. 2013), and it is possible that these phosphorylation changes are maximal. It is, therefore, also possible that lower doses of phenylephrine may result in submaximal eNOS phosphorylation changes that may allow for observation of flow‐induced eNOS phosphorylation changes. Regardless of this limitation, our study did demonstrate that a NOS‐dependent vasodilation response can occur during flow without further Ser1179 and Thr495 phosphorylation changes.

## Conclusions

This study indicates that NOS‐dependent flow‐induced dilation does not require changes in eNOS phosphorylation at Ser1179 and Thr495 beyond those induced by preconstriction with phenylephrine. This means that eNOS is activated by another mechanism/s during flow‐induced dilation in intact, preconstricted mouse mesenteric arteries.

## Conflict of Interest

None declared.

## Supporting information




**Figure S1.** Mesenteric artery dilation responses to flow in the presence of 1% or 3% BSA perfusion buffer without (A) or with (B) L‐NAME (10^−4^ mol/L). **P <* *0.01 compared to 3% BSA – L‐NAME group (two‐way ANOVA). (C) That incubation of vessels with L‐NAME in only the superfusate did not result in differential dilation responses to flow than when it was added to both superfusate and perfusate.
**Figure S2.** Dilation response of control mesenteric arteries to flowrates up to 120 μL/min. Each flow step was maintained for 5 min before diameter measurement.
**Figure S3.** Individual values of pSer1179eNOS/total eNOS for each blot included in Figure 2A. *Significantly greater (*P* < 0.05) compared to “No Treatment” (Student's *t*‐test). One‐way ANOVA did not show any significant differences between the groups.
