# Supplementary material for: Flow does not alter eNOS phosphoryation at Ser1179 or Thr495 in preconstricted mouse mesenteric arteries
Source: Physiol Rep. 2018 Sep 10;6(17):e13864. doi: 10.14814/phy2.13864 (PMC6129772; doi:10.14814/phy2.13864)
Supplement: Supplementary file 1 — Figure S1. Mesenteric artery dilation responses to flow in the presence of 1% or 3% BSA perfusion buffer without (A) or with (B) L‐NAME (10−4 mol/L). **P < 0.01 compared to 3% BSA – L‐NAME group (two‐way ANOVA). (C) That incubation of vessels with L‐NAME in only the superfusate did not result in differential dilation responses to flow than when it was added to both superfusate and perfusate. Figure S2. Dilation response of control mesenteric arteries to flowrates up to 120 μL/min. Each flow step was maintained for 5 min before diameter measurement. Figure S3. Individual values of pSer1179eNOS/total eNOS for each blot included in Figure 2A. *Significantly greater (P < 0.05) compared to “No Treatment” (Student's t‐test). One‐way ANOVA did not show any significant differences between the groups. [file PHY2-6-e13864-s001.docx]

SUPPLEMENTARY FIGURES

**Supplementary Figure 1**. Mesenteric artery dilation responses to flow in the presence of 1% or 3% BSA perfusion buffer without (A) or with (B) L-NAME (10^-4^ M). ** P<0.01 compared to 3% BSA – L-NAME group (two-way ANOVA)**.** Panel (C) indicates that incubation of vessels with L-NAME in only the superfusate did not result in differential dilation responses to flow than when it was added to both superfusate and perfusate.

A B


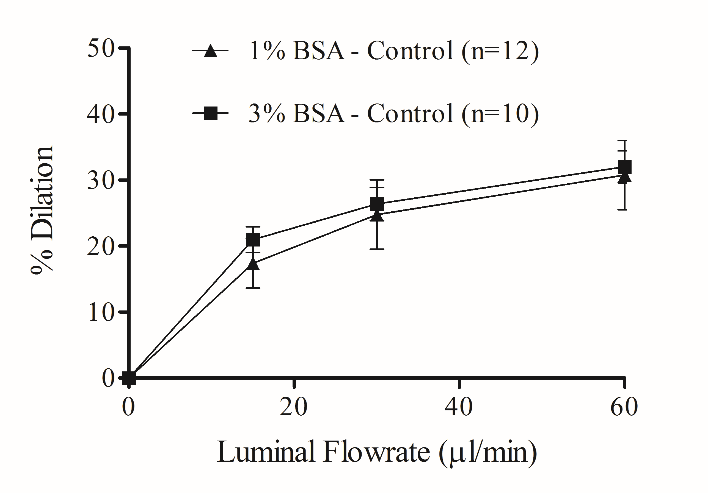

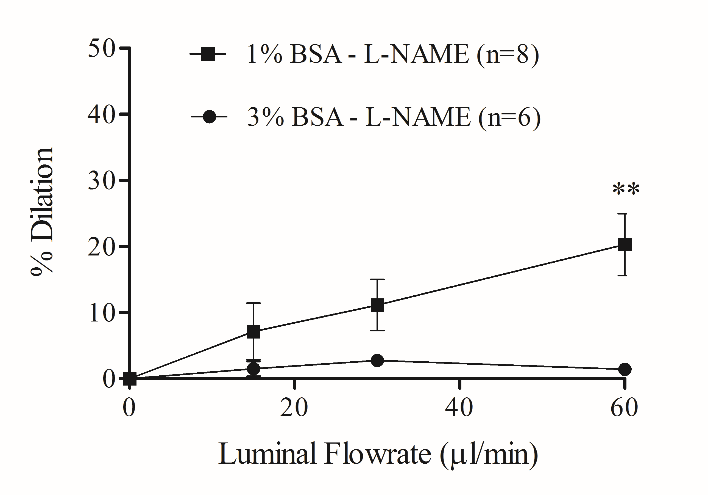


C


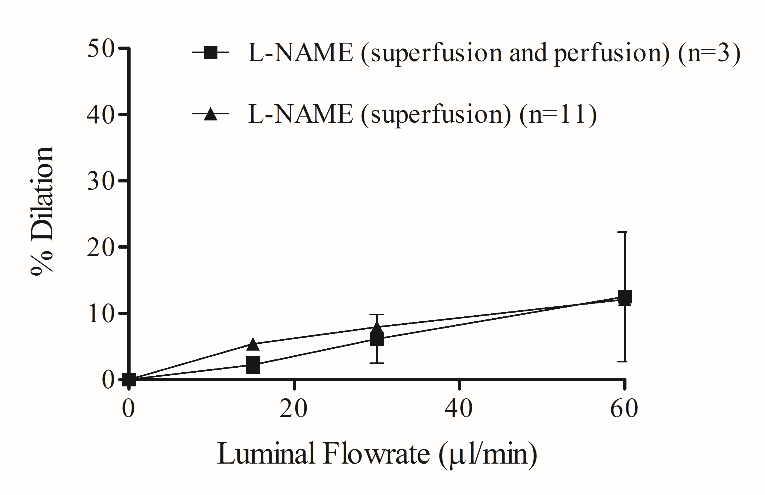


**Supplementary Figure 2**. Dilation response of control mesenteric arteries to flowrates up to 120 μl/min. Each flow step was maintained for 5 min before diameter measurement.


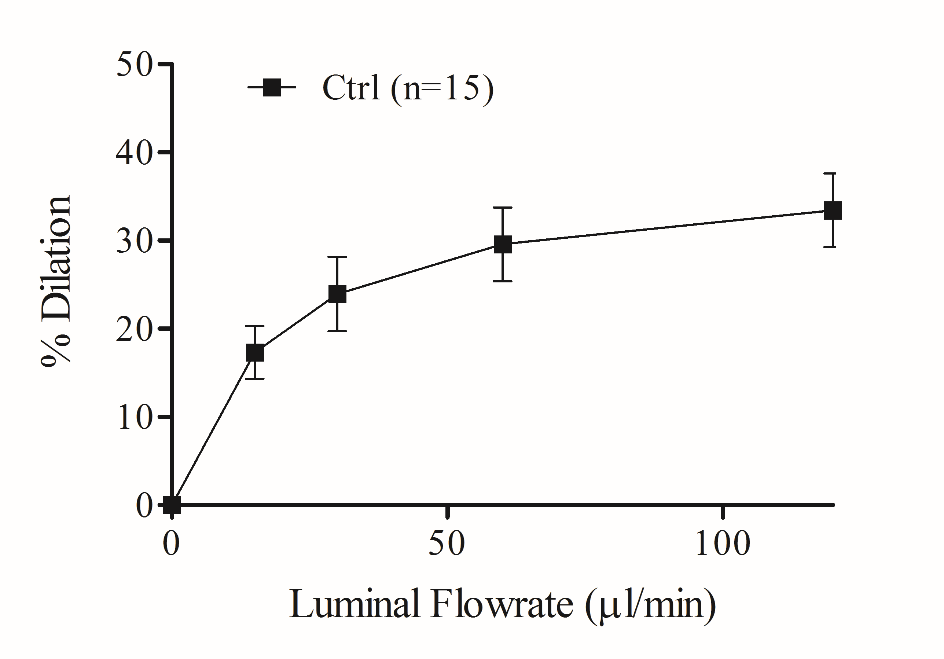


**Supplementary Figure 3**. Individual values of pSer1179eNOS/total eNOS for each blot included in Figure 2A. * Significantly greater (P<0.05) compared to “No Treatment” (Student’s t-test). One-way ANOVA did not show any significant differences between the groups.


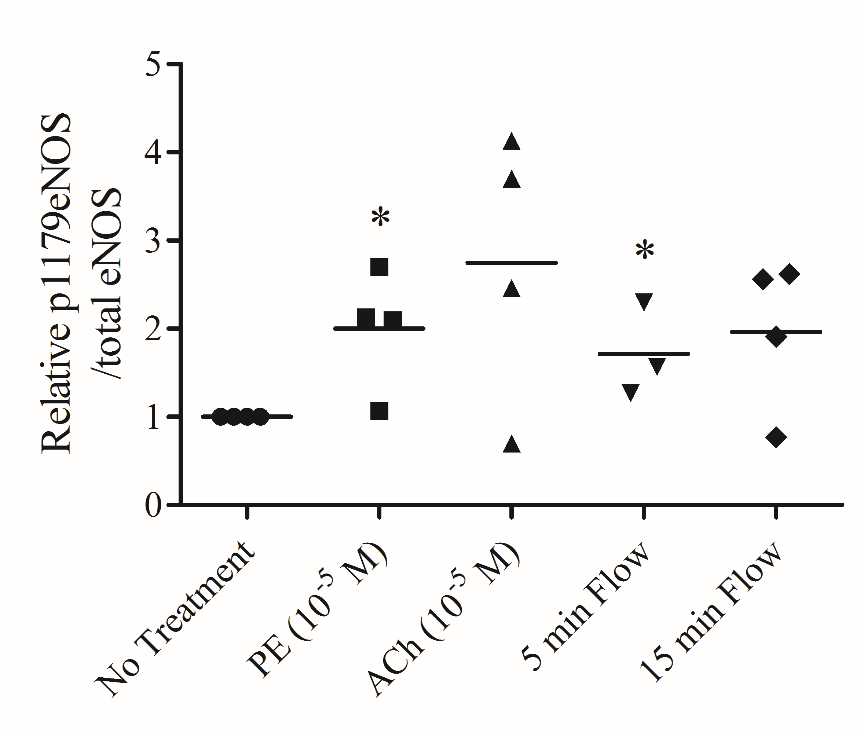


**Supplementary Figure 4**. Individual immuno-blot films included in Figure 2. Sample Sets 1-3 have four vessels pooled per treatment. Sample Set 4 contains 6 pooled vessels per treatment. “No T” is no treatment time control, “PE” is 10 min with 10^-5^ M phenylephrine, “Ach” is 10 min with 10^-5^ M phenylephrine followed by 5 min with 10^-4^ M acetylcholine, “5” is 10 min with 10^-5^ M phenylephrine followed by 5 min of 60 μl/min flow, and “15” is 10 min with 10^-5^ M phenylephrine followed by 15 min of 60 μl/min flow. Immunoblots for pSer1179eNOS and total eNOS were performed first, and remaining protein was used to perform blots for pThr495eNOS and total eNOS. This required pooling together protein from Sample Sets 2 and 3 to generate enough protein to perform the first pThr495eNOS/total eNOS blot. Responses of vessels to each treatment for each sample set are shown in Table 3. Quantification of each band (band density) was performed (in triplicate and averaged) using ImageJ (NIH) and is shown beneath each band, and normalized ratio of phosphorylated/total eNOS is shown below each sample set.

**pSer1179eNOS/total eNOS**

**Sample Set 1**

No T PE ACh 15

pS1179eNOS
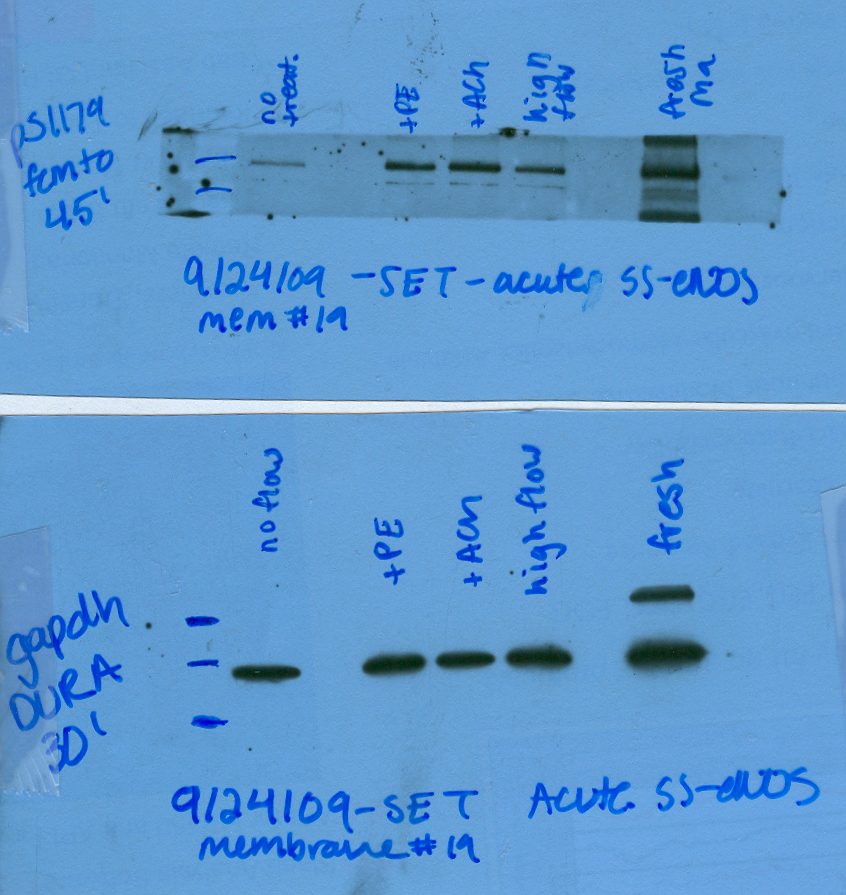


2011 5706 7821 5118

eNOS
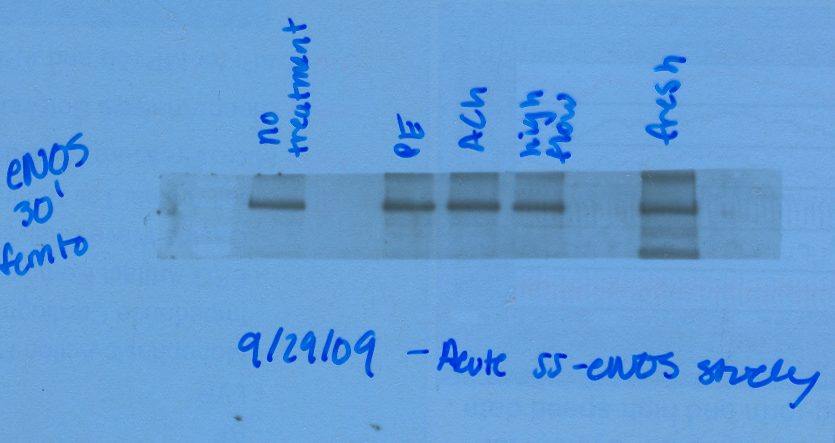


5960 6256 6264 5923

*Normalized ratio: 1.00 2.70 3.70 2.45*

**Sample Set 2**

No T PE ACh 5 15

pS1179eNOS
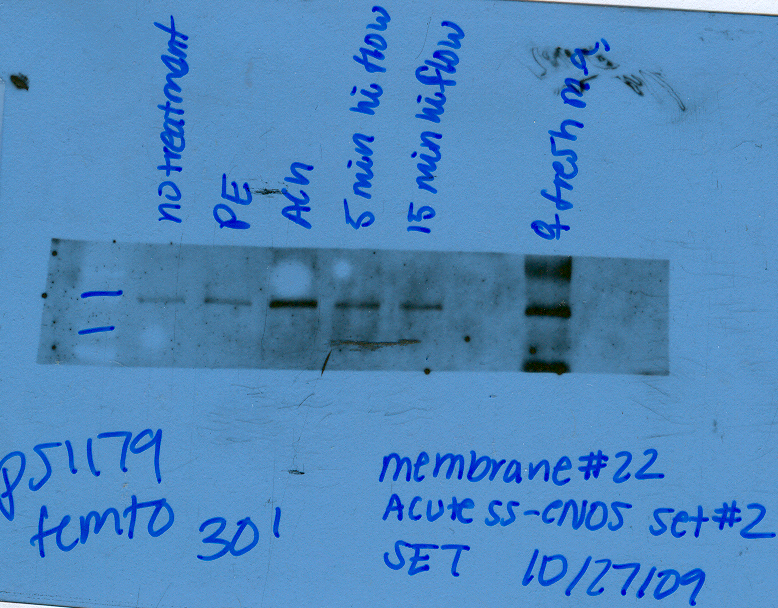


1147 1768 6742 2841 2384

eNOS
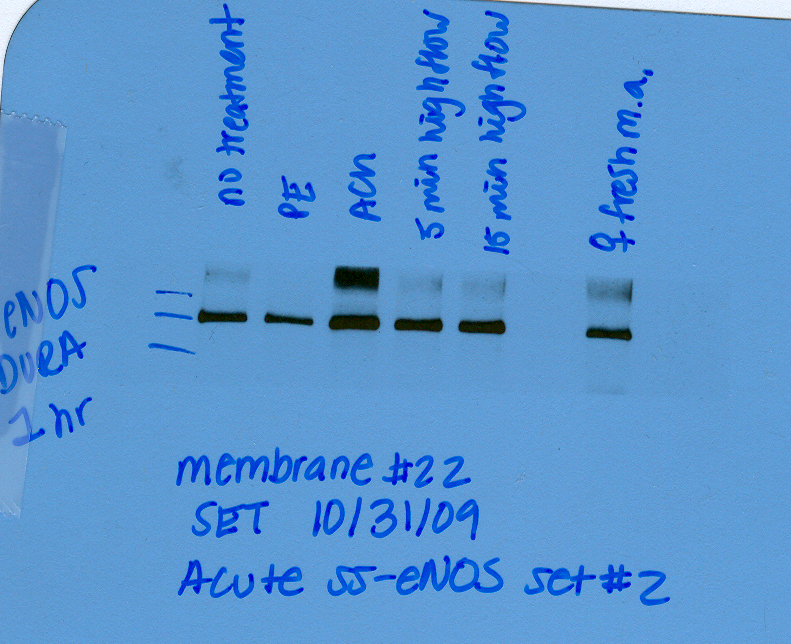


10391 7522 14795 11194 11320

*Normalized ratio: 1.00 2.13 4.13 2.30 1.91*

**Sample Set 3**

No T PE ACh 5 15

pS1179eNOS
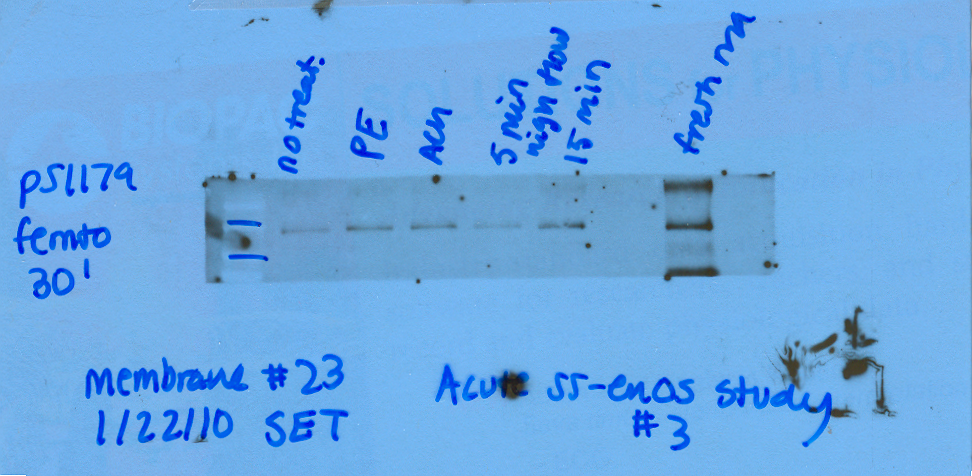


1642 2840 2928 1433 4280

eNOS
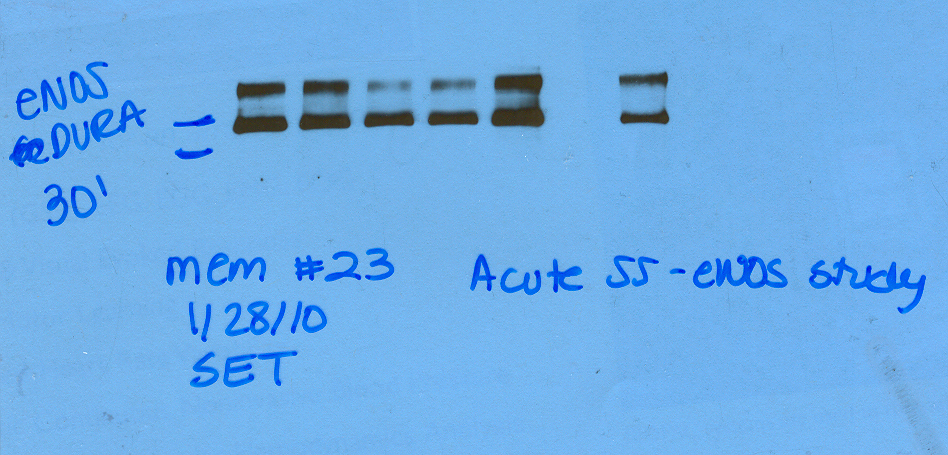


17873 14699 12972 12245 17774

*Normalized ratio: 1.00 2.10 2.46 1.27 2.62*

**Sample Set 4**

No T PE ACh 5 15

pS1179eNOS
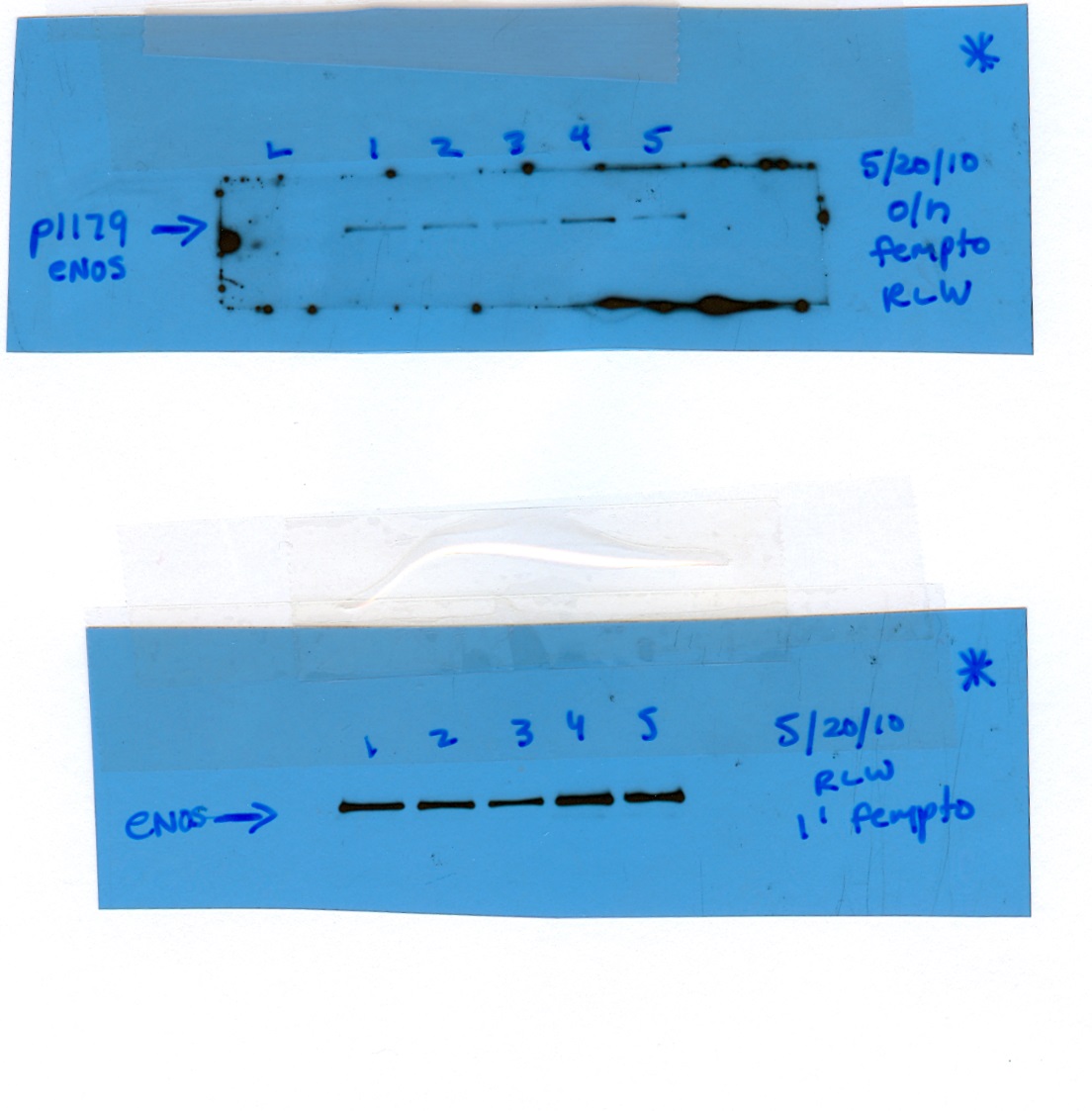


4424 3468 1827 7854 3545

eNOS
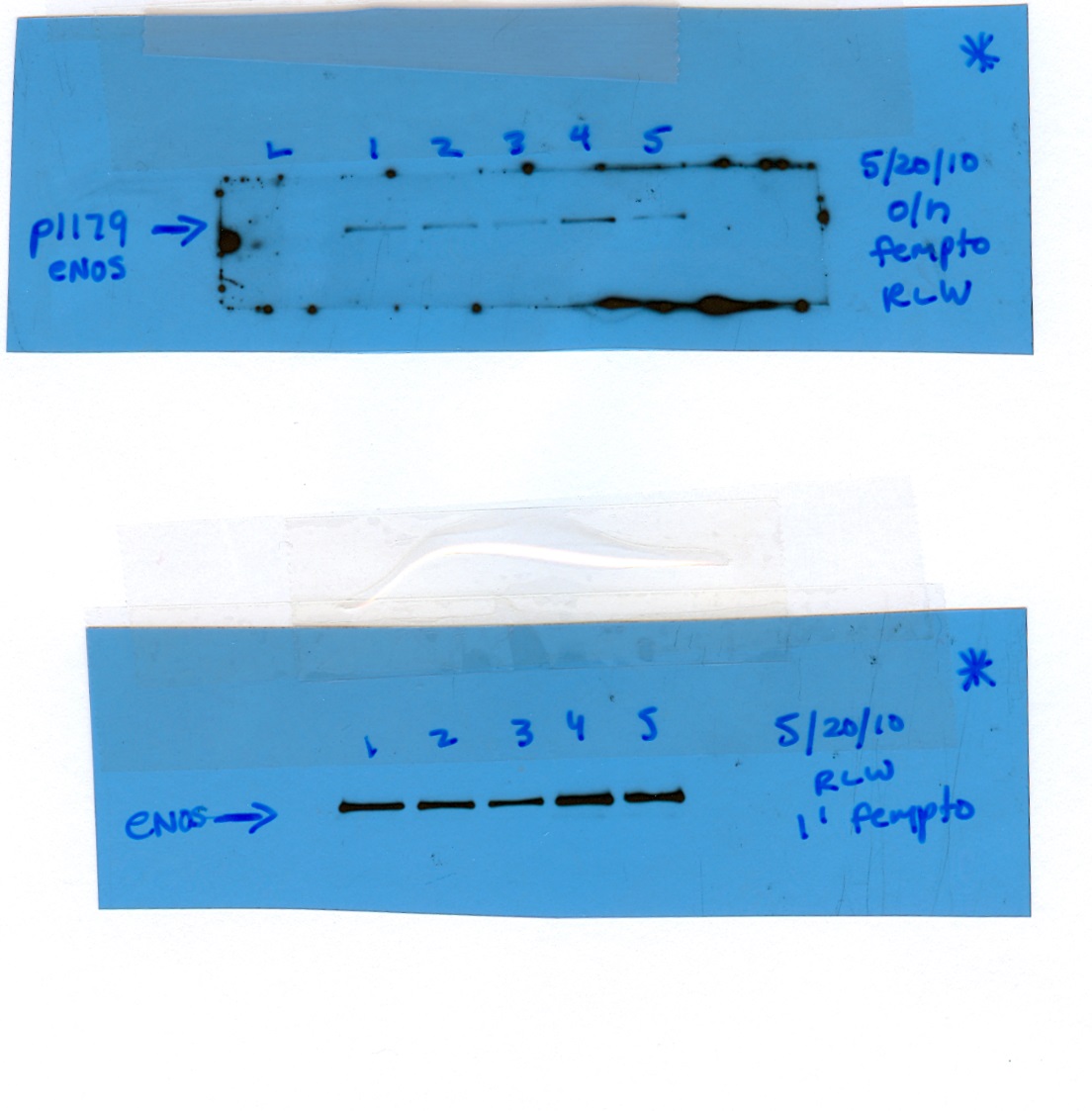


12464 9115 7323 14137 12937

*Normalized ratio: 1.00 1.07 0.70 1.57 0.77*

**pThr495eNOS/total eNOS**

**Sample Set 2&3**

No T PE ACh 5 15

pT495eNOS
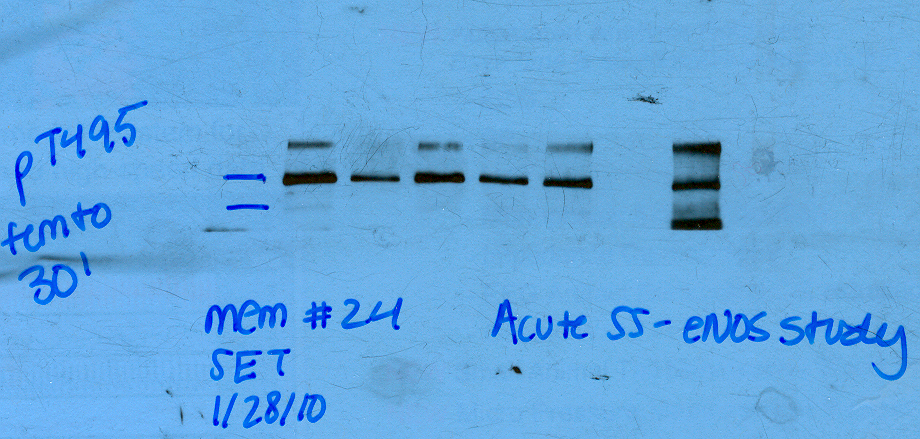


15164 5993 12794 7421 10512

eNOS
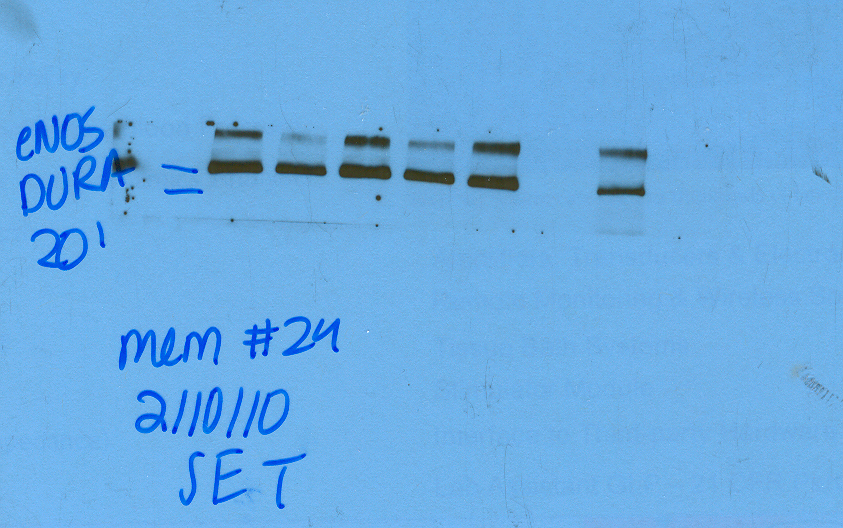


14600 11520 14462 11593 13016

*Normalized ratio: 1.00 0.50 0.85 0.62 0.78*

**Sample Set 4**

No T PE ACh 5 15

pT495eNOS
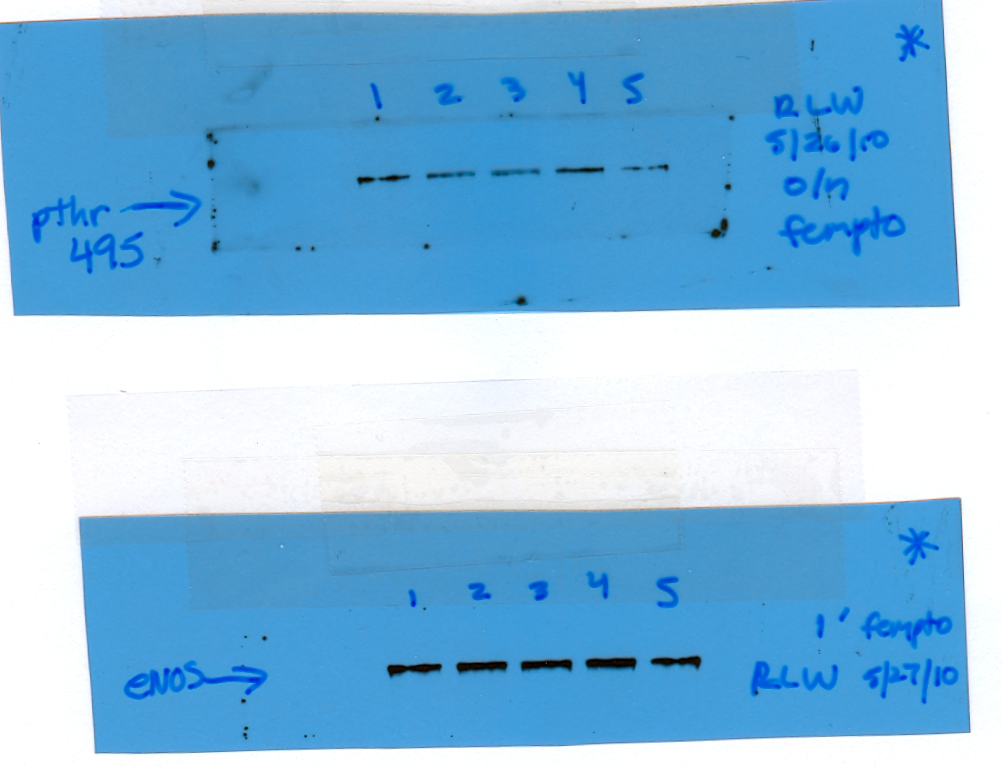


5682 3335 3017 4757 2296

eNOS
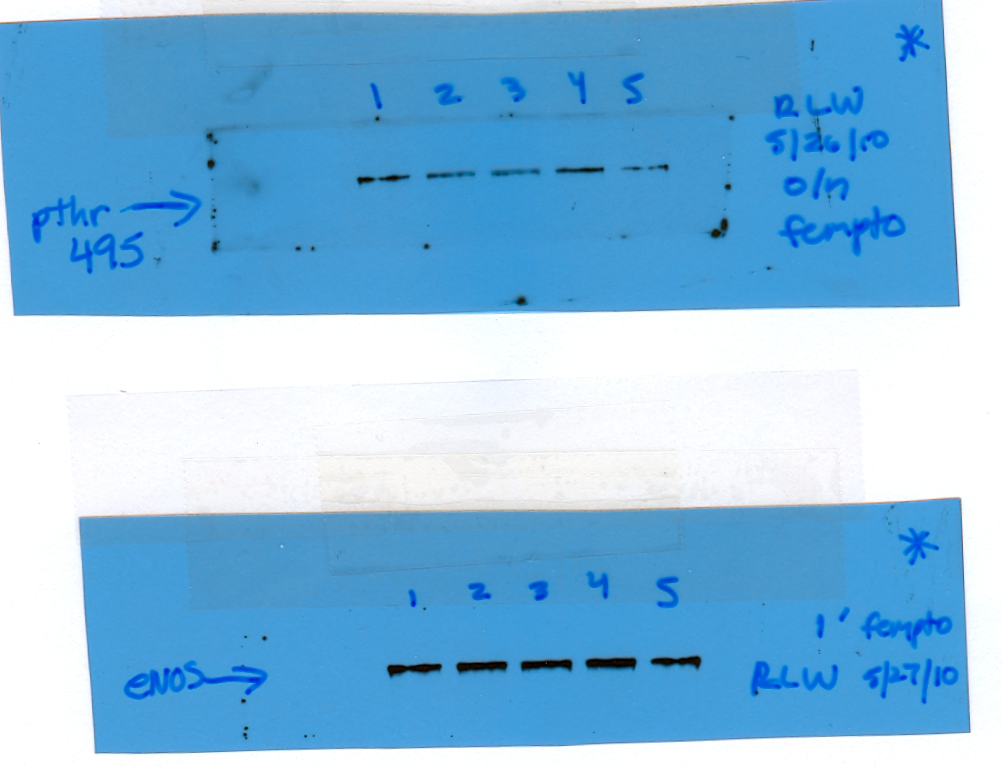


8123 8225 8143 9147 7119

*Normalized ratio: 1.00 0.58 0.53 0.74 0.46*
